# Moisture Determination for Fine-Sized Copper Ore by Computer Vision and Thermovision Methods

**DOI:** 10.3390/s23031220

**Published:** 2023-01-20

**Authors:** Dariusz Buchczik, Sebastian Budzan, Oliwia Krauze, Roman Wyzgolik

**Affiliations:** Department of Measurements and Control Systems, Faculty of Automatic Control, Electronics and Computer Science, Silesian University of Technology, 44-100 Gliwice, Poland

**Keywords:** moisture, particle size, computer vision, thermovision, modeling, copper ore, powders, grinding

## Abstract

The moisture of bulk material has a significant impact on the energetic efficiency of dry grinding, resultant particle size distribution and particle shape, and conditions of powder transport. This research aims to develop computer vision and thermovision techniques for the on-site estimation of moisture content in copper ore, for use, e.g., in dry grinding installations. The influence of particle size on the results of moisture estimation is also studied. The tested granular material was copper ore of particle size 0–2 mm and relative moisture content of 0.5–11%. Both vision and thermovision images were taken at standard and macro scales. The results suggest that median-intensity vision images monotonically reflect copper ore moisture in the range of about 0.5–5%. Suitable models were identified and cross-validated here. In contrary, thermograms should not be analyzed simply for their mean temperature but treated with computer vision processing algorithms.

## 1. Introduction

Copper is an essential material in numerous industries and the third most used metal in the world, after iron and aluminium [1]. It is widely used in many applications in industry as well as in healthcare [1,2,3]. Since the beginning of the 20th century, the use of refined copper has grown by approximately 3.4% annually—from less than 0.5 million tonnes in 1900 to nearly 25 million tonnes in 2020. This means that copper consumption has doubled in the last 25 years and more than tripled in the last 50 years [4,5]. The demand for copper is supposed to continue growing in the coming years, due to the need to transition to low-carbon energy and transportation systems [4,6]. The great majority of this demand is—and will be—satisfied by refining newly mined copper. This primary production accounted for 70% of global copper consumption in 2019 (the remaining 30% came from the recycling of copper scrap) [4]. Taking into account low ore grades—on average 0.65% copper content in the material mined in 2015 [7], which is shown to be decreasing [8]—colossal quantities of rock need to be mined, crushed, ground and then physicochemically processed to obtain the necessary amounts of pure metal.

Comminution processes are responsible for a large amount of the energy consumption in metal production [9]. For example, particles subjected to flotation should be tens of micrometers in diameter [10] and should make up a flour-like powder, is achieved over several stages of crushing and grinding. Of course, this leads to continuous industrial and scientific efforts to provide more efficient grinding techniques. One of the emerging solutions in the field is the use of electromagnetic mills [11,12], especially in conjunction with a complete grinding and classification circuit with the pressured pneumatic transport of the processed material [13,14]. The installation is equipped with a dedicated measurement system and hierarchical automatic control system, as detailed, e.g., in [13,15]. It is suitable for the ultrafine grinding of feed material sized up to 1–2 mm, depending on the mill size.

In general, grinding processes, and also the discussed installation with an electromagnetic mill, are strongly affected by moisture content in the raw material, in several ways [16,17]. The amount of water in the working chamber of the mill defines the residence time necessary to achieve the desired degree of fineness (so, moisture impacts grinding efficiency). The resultant particle shapes also differ with moisture level during grinding. Some constraints on the final product’s moisture may come from the technological usability of the material, from transport and storage methods, durability (shelf life), etc. Additionally, powder transport is moisture-limiting in the grinding circuit itself: powders that are too damp do not move freely in the pipeline and stick to the elements, whereas powders that are too dry might explode (unless specialist gases are used instead of air).

As a result, material moisture needs to be monitored throughout the grinding installation. It should be measured where possible (e.g., in fresh feed, in the final product, and in the material stream recycled to be reground, if present); modeling when taking measurements is impossible or very difficult and costly. These locations include the working chamber of the mill, particle classifiers (if present), and transport pipes. Some moisture modeling issues in electromagnetic mill installation were addressed in [16,17]. On the other hand, there are the moisture measurements that are used directly or as inputs to the mentioned models. The most popular non-contact methods used in industry involve microwave or radiometric sensors. Their biggest advantage is the possibility of taking non-contact measurements of materials in motion, e.g., transported on conveyor belts. However, these sensors are complex and very costly. Contact capacitive moisture sensors are much simpler and cheaper. Their disadvantage is the need to use electrodes that are in contact with the measured material, which in the case of moving material causes additional errors and the rapid mechanical wear of the electrodes. The demand for moisture measurements led to new techniques being proposed by a research group researching electromagnetic mill installation. These include the patented contact method based on combined measurements of surface impedance and near-infrared absorption [18,19,20], or the contactless vision and thermovision methods investigated here.

Firstly, in the current paper, the authors tried to utilize typical, i.e., the most popular, IR cameras, that is LWIR (Long Wavelength InfraRed) cameras. IR temperature measurements are based on Planck’s law of the distribution of blackbody radiation. This is widely described in the literature, and details may be found in many books dedicated to IR thermal imaging or chapters in books on temperature measurements, e.g., [21]. The main problem with IR temperature measurements is the emissivity coefficient. It is equal to 1 only for blackbodies, which are perfect emitters and absorbers of radiation. However, they do not exist in practice. For realbodies, the emissivity is between 0 and 1 and generally depends on the wavelength and temperature [22]. A very important factor is also the surface of the material whose temperature is being measured. For example, a polished metal surface is highly reflective, so the emissivity is very low (closer to 0, e.g., 0.1), while matt and porous surfaces have a higher emissivity (closer to 1, e.g., 0.9). Moreover, for any real surface, the radiance usually also depends on the angle of emission; however, this dependence is less for higher-emissivity surfaces. In the research presented here, the amount of surface moisture was expected to affect the temperature indicated by the camera.

Secondly, the authors have experience in using vision methods to assess the degree of grinding of copper ore [23,24]. The publications used segmentation algorithms with modification for the incorrect detection of particles. In [24], a number of modifications were applied to combine two classical approaches to the detection of copper ore particles, namely regions with growing segmentation and edge detection, where region growth was limited only to the particles boundaries. The proposed method showed that the solution based on image analysis can be used to test online materials. It can also be used for a wide range of particle sizes. On the basis of the authors’ experience, this article describes a series of experiments, the aim of which was to check the possibility of relying on image information when assessing the moisture of copper ore with a wide range of moistures and particle sizes. This would allow one to estimate multiple material characteristics from a single image—involving quantities such as moisture content (described here), particle sizes and shapes [23,24], and the active surfaces of metals in ore [25].

This paper makes the following novel contributions:

- We test fine-ground copper ore over a wide range of moistures using thermovision and vision methods;
- The development of a method for assessing the moisture content of raw materials carried out simultaneously with the optical assessment of the material to determine particle size;
- The validation of the suggested relationships between material moisture and image intensity.

This paper is organized as follows: Section 2 reviews previous studies related to the measurement of material moisture with vision and thermal images. Section 3 describes the processing of raw material and the details of the methods are provided here. The experimental results on thermal and vision images are presented and discussed in Section 4. Finally, the conclusions are given in Section 5.

## 2. Related Work

In [26], the authors summarize sensing methods for grain moisture measurement, including the methods and techniques developed over the last 15 years, based, e.g., on radio frequency, hydrogen nuclear magnetic resonance and visible spectrum analysis. NIR (Near-InfraRed) has been used for many years for the measurement of leaf water content [27]. The NIR wavelength ranges from 0.76 to 2.5 µm. The advantage of using the NIR wavelength spectrum to measure surface moisture is that a number of wavelengths have strong moisture absorption capabilities [28,29,30]. In [16,19], the NIR approach is described for moisture measurements in the grinding process with an electromagnetic mill. The authors state that this method provides good results, especially when there are very low water contents in the sample. However, no newly designed device has been available for measuring copper ore moisture in the NIR range until now. In [30], a measurement method is proposed to simultaneously characterize the moisture content and size of individual pharmaceutical granules. The authors used the combination of NIR spectroscopy and image analysis to solve this problem.

Another method used to determine the degree of moisture in a material is image processing. The literature presents applications of vision methods to assess the moisture of soil [31,32], milled rice [33], loess [34], coal [35], and iron ore [36,37]. In [31], the images were processed to determine the median values in the red, green, and blue bands, as well as the hue, saturation, and value of the HSV color space. Finally, digital numbers of a panchromatic image were obtained. In [33], the authors tested a few types of rice.The method involved image processing with a perceptron neural network algorithm. The authors of [34] proposed a mathematical model to describe the characteristic curve of water content vs. gray value, which was based on RGB values. In [35], the authors proposed an algorithm for the online testing of coal. In their solution, it was also possible to determine the size of the particles. The algorithm was based on functions of MATLAB software, i.e., morphological operations, distance transformation, and watershed segmentation. A novel neural network model based on an attention mechanism and bidirectional ResNet-LSTM structure was presented by the authors of [38]. This method was used for the monitoring of coal moisture content. A review of selected methods of soil moisture evaluation can be found in [39]. The authors raised the issue of different types of images in the analysis: satellite images, unmanned aerial vehicles images, hyperspectral images, thermal images, and digital images. As part of the review, they also drew attention to selected methods, primarily the analysis of selected areas of interest and the RGB and HSI color spaces.

## 3. Materials and Methods

### 3.1. Raw Material

**Figure 1:**
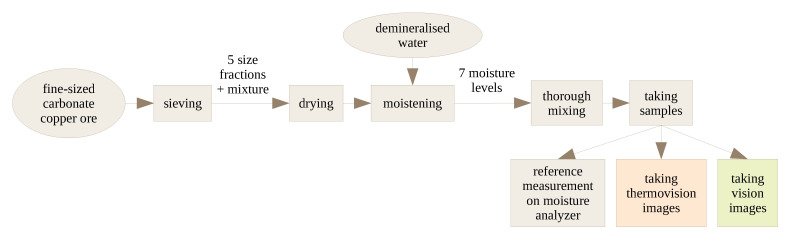
Stages of processing of the raw material during the experiment.

**Figure 2:**
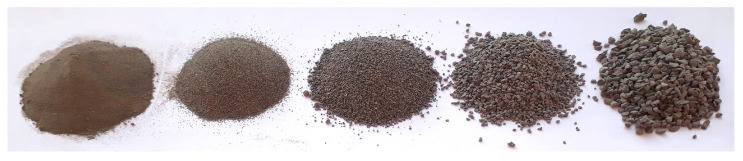
Dry samples of all individual size fractions of copper ore used in the experiments. From left to right: particles of size 0–0.1 mm; 0.1–0.2 mm; 0.2–0.5 mm; 0.5–1 mm; 1–2 mm.

**Figure 3:**
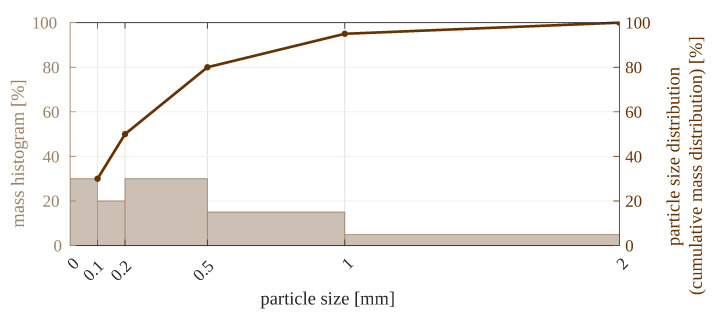
Composition of the mixed-size material used in the experiments.

The treatment of raw material during the experiment is summarized in Figure 1. The material was carbonate copper ore, with particles sized 0–2 mm. It was the product of grinding in a ball mill, which is a usual stage in ore processing. Such material is suitable to be fed to next-stage mills, such as the electromagnetic mill, for fine or ultrafine grinding. The particles were separated into several size fractions using an air jet sieving machine or a sieve shaker, depending on the experiment series. The following material fractions were used in the experiments (the sizes given are sieve mesh sizes, so they reflect particle diameters): 0–0.1 mm; 0.1–0.2 mm; 0.2–0.5 mm; 0.5–1 mm; 1–2 mm; and a mixture thereof. All the five individual size fractions are visualized in Figure 2, and the composition of the mixture is detailed in Figure 3. The content of each size fraction in the mix was chosen such that the whole blend resembled the actual feed for the electromagnetic mill, similar, e.g., to the raw material used in [17].

It must be noted that each fraction contained some small amount of undersized particles. This is because carbonate copper ore is a relatively soft rock and it slightly rubs to dust during handling.

**Figure 4:**
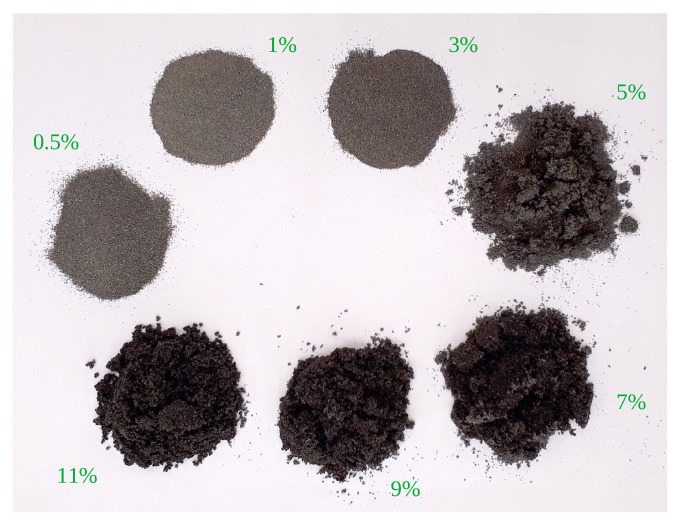
Samples of 0.1–0.2 mm copper ore at different moisture levels.

The tested materials were oven-dried at a temperature of about 110–120 °C. The dry materials exhibited relative moisture contents of (1) about 0.3–0.5%, depending on particle size. Samples of these dry powders were used in the experiments, alongside more moistened samples. The latter were produced through a thorough mixing of the material with demineralized water, in amounts suitable to achieve relative moistures of around 1, 3, 5, 7, 9 and 11%. Samples of moistened material are shown in Figure 4. The exact moisture content measured in the material is listed in Supplementary Materials, Tables S1 and S2. Thus, the experiment involved six granulations of copper ore at seven moisture levels each.

The moisture range adopted in this study was possibly broad, as the goal was to determine the applicability of the tested methods at various moisture levels. In fact, ore having 11% relative water content was wet enough for the moisture to start gathering at the bottom of the container, especially with the coarser particles. This means that free moisture—i.e., that not adsorbed on the particles’ surfaces—started to occur [40]. Nearly continuous mixing was then needed to keep the sample homogeneous, and images had to be taken immediately after collecting the sample. This is practically the upper limit of moisture content that could be tested; thus, at this point, more wet material would already be a slurry, not a moistened granular material. On the other hand, the mid-range moisture of 5% resulted in copper ore consistency like this for damp sand used for building castles in a sandbox.

### 3.2. Reference Moisture Measurements

To precisely verify material moisture during the experiment, the Radwag MA 110.R moisture analyzer was used [41]. This device combines precise balance and a heating chamber. The relative (i.e., wet-basis) gravimetric moisture content MC is defined as the ratio of mass loss due to heating (mass of evaporated water) to the mass of the wet sample:

(1) $$MC=\frac{m_{\mathrm{water}}}{m_{\mathrm{wet}}}\cdot 100\%=\frac{m_{\mathrm{wet}}-m_{\mathrm{dry}}}{m_{\mathrm{wet}}}\cdot 100\%\;,$$

with *m* denoting mass and the subscripts "water", "wet" and "dry" indicating the contained water, wet (moist) material and completely dried material, respectively.

Material samples used for reference moisture measurements weighed about 7–9 g (10–12 g for the most coarse fraction). Other material samples were used for taking vision and thermovision images, as described in the following sections.

According to the moisture analyzer’s manufacturer [41], measurement repeatability for samples of the masses used is ±0.01%. Moreover, the paper [19] states that standard uncertainty type A due to material sampling is 0.05% for homogeneous material in the tank, and 0.15% for not fully homogeneous material. The former case is easy to achieve with low to moderate moisture; the latter occurs for high moisture, especially for coarse particles.

Some scientific fields or industry branches use volumetric moisture content instead of the gravimetric (mass) one. This was not used in the present research as it would be harder to take reliable reference measurements to determine the volumetric moisture content; in contrast, mass moisture content is determined directly by the moisture analyzer. However, it is worth mentioning this topic to provide the readers of such research with a better context.

For the wet-basis measurements (with the wet material’s features used in the denominator)—as used so far—volumetric moisture content $MC_{\mathrm{vol}}$ is defined as:

(2) $$MC_{\mathrm{vol}}=\frac{V_{\mathrm{water}}}{V_{\mathrm{wet}}}\cdot 100\%=\frac{\frac{m_{\mathrm{water}}}{\rho_{\mathrm{water}}}}{\frac{m_{\mathrm{wet}}}{\rho_{\mathrm{wet}}}}\cdot 100\%=\frac{\rho_{\mathrm{wet}}}{\rho_{\mathrm{water}}}\cdot MC\;,$$

where *V* denotes volume and ρ is density. Bulk volume and bulk desity are considered in the case of the material, that is, inter- and intra-particle voids are also counted [42]. Metal ores are much more dense than water; the Polish carbonate copper ore used in the presented experiments has a dry bulk density of approximately 2.6 tonne/m^3^ [42]. Thus, for this material, the volumetric moisture content differs significantly (in terms of numerical values) from the mass moisture content; namely, the former is around 2.6 times higher than the latter. MC=0.5% corresponds roughly to $MC_{\mathrm{vol}}=\left[1\ldots 1.5\right]\%$, MC=5% to $MC_{\mathrm{vol}}=13\%$, and MC=11% to $MC_{\mathrm{vol}}=\left[28\ldots 29\right]\%$.

### 3.3. Thermovision Images

**Figure 5:**
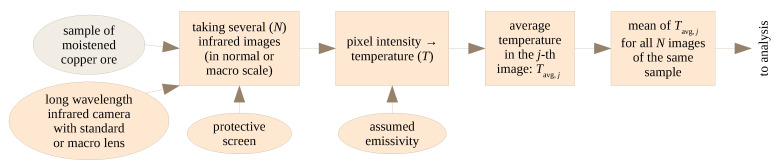
Stages of acquisition and processing of thermovision images.

The thermovision images were acquired and processed according to the flowchart in Figure 5. Two measurement set-ups were used to capture thermovision images. In the first approach, the IR camera was a FLIR A325 with dedicated Close-up Lens 1x. In the second approach, the FLIR A325 camera with standard, build-in optics was used. In all experiments, the camera was switched on long before the measurements were taken, to ensure stable temperature readings. Flir Tools software was used for image acquisition and then IR image processing. FLIR A325 is a LWIR (Long Wavelength InfraRed) camera, and measures the IR temperature in the 7.5 to 13 µm spectral range. The resolution is 320 × 240 pixels and the noise equivalent temperature difference (NETD) is equal to or less than 50 mK. The temperature measurement accuracy is ±2 °C or ±2% of the reading (which is greater), although this is not important in the present case, as here the point is the difference in the surface temperatures of different samples.

**Figure 6:**
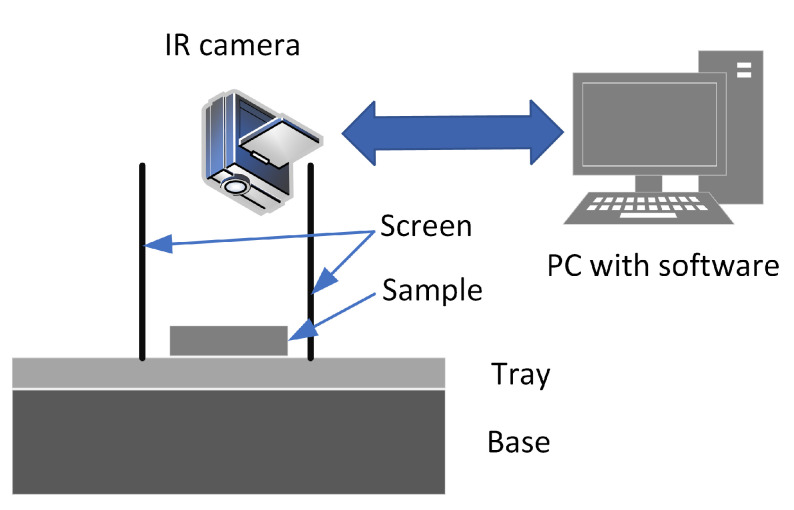
Measurement set-up for IR images acquisition.

**Figure 7:**
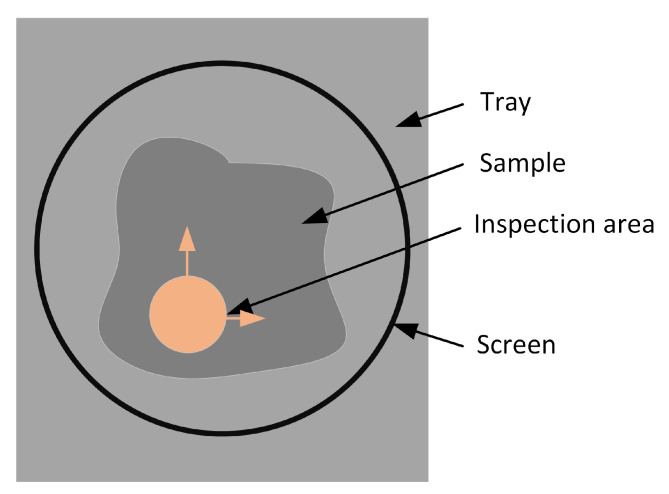
IR image acquisition from an ore sample for the first approach (with macro lens).

A screen was used to prevent radiation from external sources (see Figure 6). The lens used in the first approach had very high magnification, so the IR image was taken only from a small part of the ore sample. To average the measurements, a set of images were acquired from different areas of the sample, as shown in Figure 7. A total of 260 thermograms of copper ore particles was recorded for the first approach and 140 for the second.

The structure of copper deposits occurring within the Lubin–Glogow district in Poland is diversified [43,44]. Among others, in the copper-bearing series there are sandstone and carbonate rocks, the latter dominated by dolomites. In the available emissivity tables, the emissivity coefficient for sandstone is rated from 0.59 to 0.70, while for dolomite it is about 0.9 [45]. Because the material is ground, copper ore is generally dull and has graining; the surface is porous, which increases emissivity. We assumed that the target emissivity is 0.9, so the temperature error caused by the improperly selected emissivity would change with the moisture. Higher material moisture means increased amounts of water, whose emissivity is high (0.95–0.98), and should be reflected in the temperature measurement of the copper ore sample. Moreover, the increase in humidity modifies the material structure by forming particle aggregates, which should also raise the actual emissivity of the sample surface.

With such assumptions for the emissivity value, the acquired IR images were scaled to the temperature readings. The average temperature was calculated from each image and studied further. These mean values of the measured surface temperatures are given in the Supplementary Materials, Tables S3–S6.

### 3.4. Vision Images

**Figure 8:**
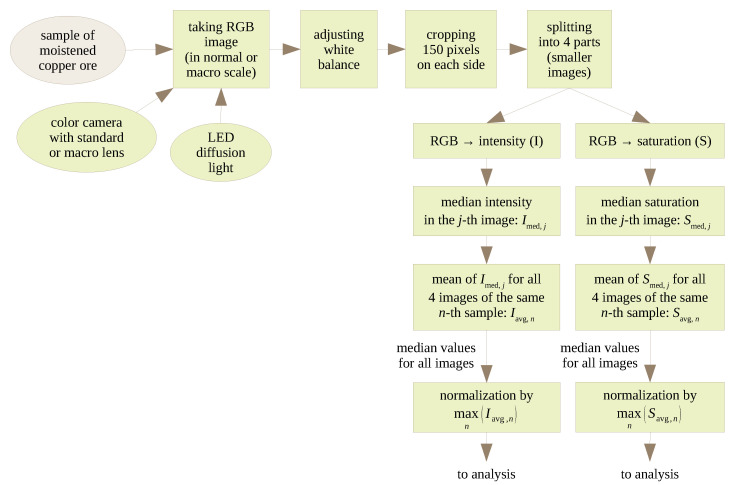
Stages of acquisition and processing of vision images.

The acquisition and processing of vision images are summarized in Figure 8. Material samples for computer vision tests were not subjected to special preparations. It was intended as a simulation of the situation in a real grinding system, in which the sample is taken directly from the pipeline by a sampler and then—most often, by gravity—it is moved to the working space of the vision camera.

**Figure 9:**
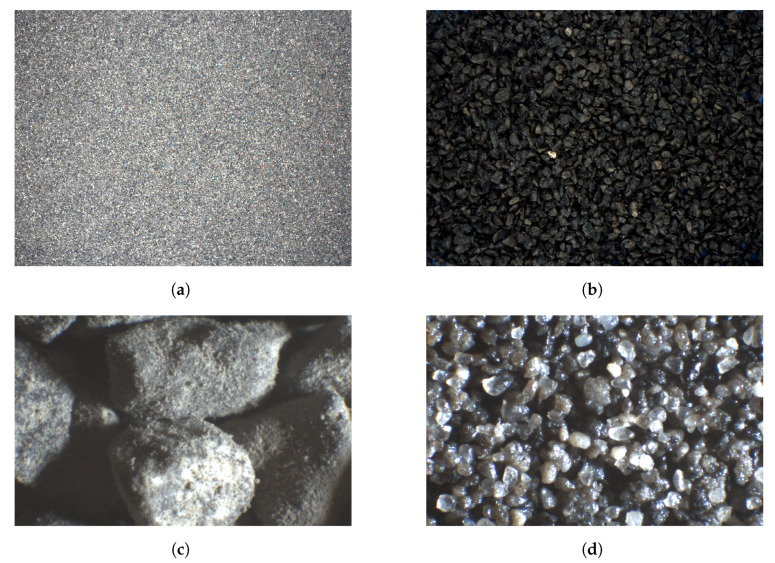
Samples of tested material: (**a**) normal mode, 0.5% moisture; (**b**) normal mode, 5% moisture; (**c**) macro image, 1% moisture; (**d**) macro image, 9% moisture.

Two types of cameras were used for different purposes. A Basler color camera with a resolution of 1624 × 1234 pixels and a 16 mm lens was used for the main tests to observe the entire surface of the test sample. A Basler color camera with a resolution of 1920 × 1200 pixels, a 50 mm lens and spacer rings was used to perform random macro tests. The macro images allowed us to observe several phenomena that are invisible with a normal lens, i.e., the formation of particle aggregates, and the brilliance of particles at specific moisture contents. The tested samples were illuminated with an LED diffusion light, whose task was to uniformly illuminate the entire surface of the sample. Nevertheless, white balance was used to normalize the color of the images for all particle sizes. Correcting the white balance ensures that the image is recorded with colors similar to those of the material under the specified lighting. Exemplary images taken in normal and macro modes are presented in Figure 9.

**Figure 10:**
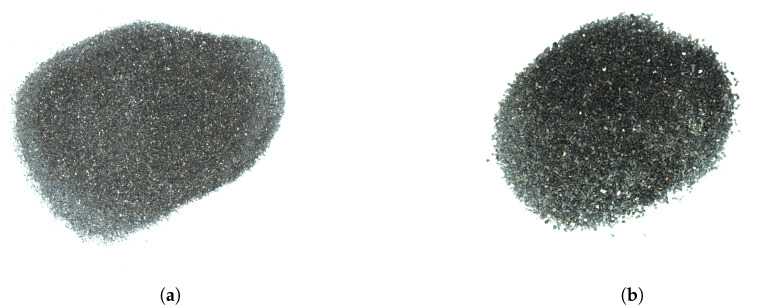
Drying copper ore at 3% moisture: (**a**) 0.2–0.5 mm particles; (**b**) 0.5–1 mm particles.

Image acquisition and analysis were carried out in a LabVIEW environment, which enables the registration, analysis and processing of digital images simultaneously. As part of the experiments, 450 images were recorded for moisture in the range of 0.5–11%, for materials of specific particle sizes and for one material mixture. Image acquisition took place immediately after placing the material in the working area of the camera. This was to avoid possible changes in material moisture during the experiment, near the edges of the sample (Figure 10). This phenomenon occurred for moderate values of material moisture: 1–5%. For this reason, the vision system was set geometrically in such a way to ensure that the image frame is completely filled with the sample of the material. In addition, the image area that was subjected to analysis was reduced by 150 pixels on each side, which gave about 1.5 million pixels in the analyzed area. This cropped image was then split horizontally and vertically into halves, resulting in four smaller images that were further analyzed separately.

As part of the image analysis, the geometric features of the particles were not assessed, which is the case when estimating particle size composition. Instead, the focus was on the assessment of parameters that can be associated with the changing moisture content of the material. Namely, the analyzed parameters were the median intensity calculated in the RGB color space and median saturation extracted from the HSL space.

The tested copper ore samples contain material of various sizes; in addition, these are irregularly shaped particles. Therefore, depending on the arrangement of individual particles, light flashes may appear (especially at higher moisture), or shadows as a result of mutual shading of particles. Therefore, it was decided to analyze the median intensity aggregated from all three (red, green, and blue) color channels, and not to analyze the independent channels. Each original RGB image was also converted to the HSL space, where colors are represented by hue, saturation and lightness. Saturation was used in the analysis as a correct measure of color purity: less gray images produce a higher value of saturation. All the values were then normalized to between 0 and 1, where 1 corresponds to maximum median value of intensity or saturation. The exact values acquired are listed in the Supplementary Materials, Tables S7–S10 (intensity) and Tables S11–S14 (saturation).

## 4. Results and Discussion

### 4.1. Thermovision Images

**Figure 11:**
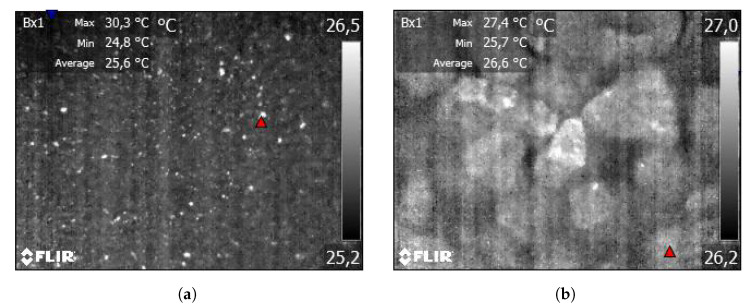
IR images in the first approach (macro images) with pure copper visible as bright spots. Ore particles of size: (**a**) 0–0.1 mm; (**b**) 1–2 mm.

Pure copper has low emissivity, even when partially oxidized, so naturally it appears as bright spots in the acquired infrared images. The copper particles reflect thermal radiation from inside the infrared camera, so their temperature is significantly higher than that of their surroundings. This is especially noticeable for small particle sizes, where there exist single copper particles (Figure 11a). For a coarser material sample, copper may constitute a small part of a particle, as shown in Figure 11b (bright parts on the particles).

**Figure 12:**
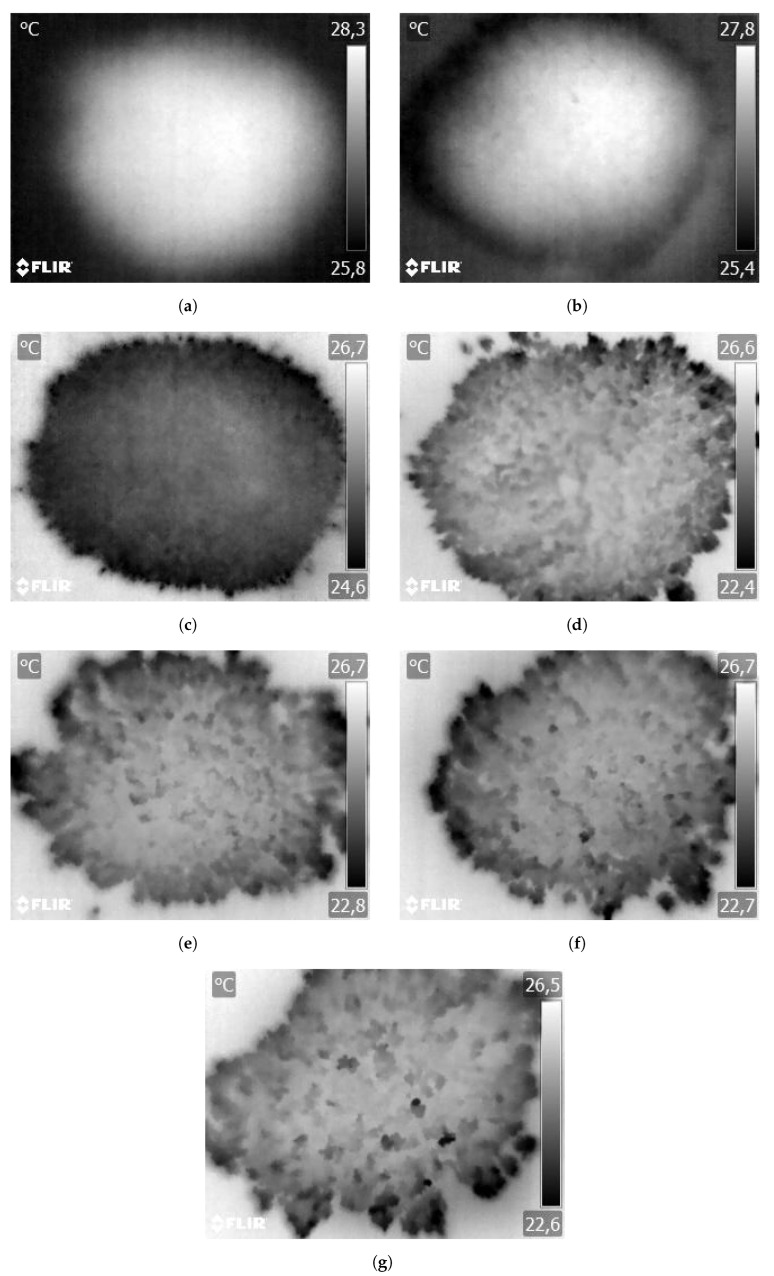
Sequence of IR images for 0.5–1 mm material with moisture contents of: (**a**) 0.5%, (**b**) 1%, (**c**) 3%, (**d**) 5%, (**e**) 7%, (**f**) 9% and (**g**) 11%.

In the case of the second approach at a large scale (whole sample was captured by the IR camera), the change in the structure of the sample surface is highly visible for the same particle size and different moistures. This is obvious due to the particles sticking together with an increase in their moisture contents. This is presented in Figure 12.

**Figure 13:**
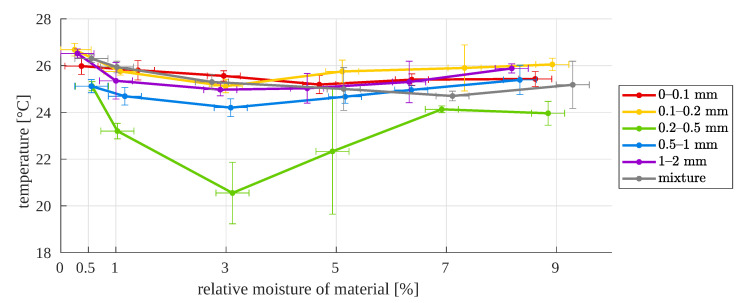
Surface temperature as a function of relative moisture, for different particle sizes—first approach (macro images).

The data were compiled in such a way as to trace the surface temperature dependencies for a given particle size distribution at varying and given (known) moisture contents of the sample. Figure 13 presents the results from macro-scale images for all investigated particle sizes and for different moisture contents of the samples. The error bars in the graph were determined as described in Appendix A.

In most cases, the lowest temperature was recorded for a relative moisture of 3%. However, the characteristics are not monotonic and, in principle, it is not possible to unambiguously relate the surface temperature of the particles to their moisture levels.

**Figure 14:**
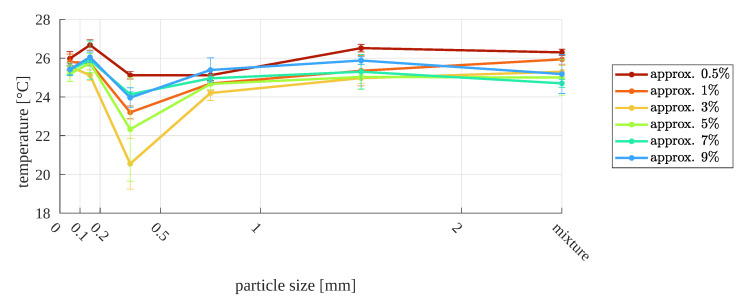
Surface temperature as function of particle size, for different relative moistures—first approach (macro images).

The second dependence, which is plotted in Figure 14, is the sample surface temperature as a function of particle size for the given sample moisture. As can be seen, there is also no clear trend, even if before the tests the authors expected that the nature of changes in relative moisture would be monotonic for each of the particle sizes.

**Figure 15:**
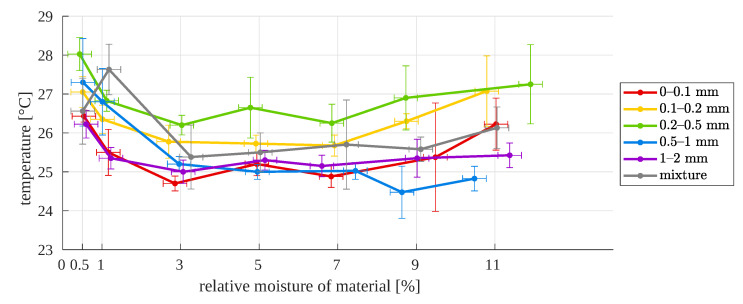
Surface temperature as a function of relative moisture, for different particle sizes—second approach (normal images).

**Figure 16:**
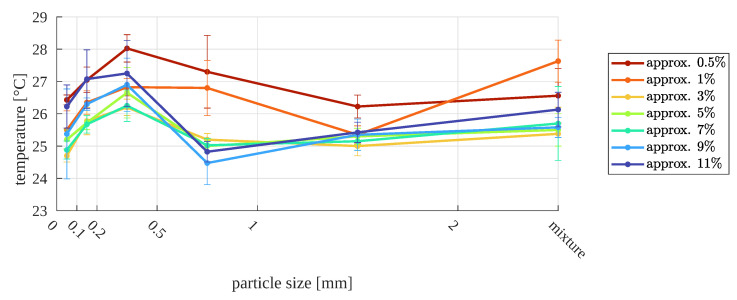
Surface temperature as a function of particle size, for different relative moistures—second approach (normal images).

For the second approach (with a standard lens), the preliminary results are also not promising (Figure 15 and Figure 16). Globally, there is no premise that enables the relationship between them to be determined, e.g., the moisture for a given particle size and surface temperature of the sample.

Taking a look at Figure 12, it seems that the image processing of thermograms might be a better approach. With the increase in the moisture of the particles, they begin to form more and more dense lumps, which could possibly be used for moisture assessment. However, this is not the aim of this article and is planned as future work.

### 4.2. Vision Images

The aim of this research is to check whether there is a relationship between the moisture of the material and the parameters of the copper ore image acquired under conditions similar to those in industry. For this reason, the results are presented in the form of collective graphs that show how image intensity or saturation depend on the moisture content, in two forms: a summary for specific particle sizes in relation to the moisture value and an additional chart for specific moisture values in relation to particle size. The graphs contain error bars, determined as described in Appendix A.

**Figure 17:**
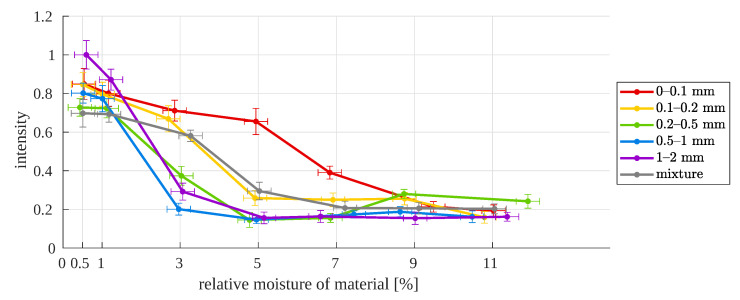
Image median intensity as a function of relative moisture for different particle sizes.

A generally observed relationship is the decrease in intensity with the increase in material moisture (Figure 17). Naturally, this manifests itself in a darkening of the image, which is caused by a decrease in the contrast between the particles. Due to their irregular shape, the boundary areas between them are important, where the material moisture affects the final results of the analysis, most often causing some anomalies; these will be described later in the discussion. The mentioned particle shape can influence the result of the analysis by changing the refraction index as well as the shine effect for larger particle sizes, which directly affects the contrast in the image.

**Figure 18:**
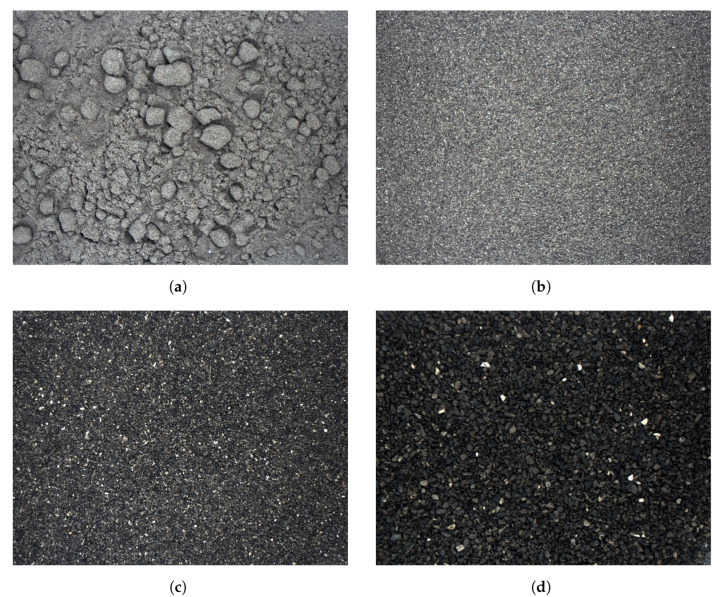
Copper ore samples of 3% moisture, with particle sizes of: (**a**) less than 0.1 mm; (**b**) 0.1–0.2 mm; (**c**) 0.2–0.5 mm; (**d**) 0.5–1 mm.

For higher values of moisture, there is a noticeable decrease in the intensity value. Additionally, the range of changes in intensity for all sizes of particles is narrowed compared to the values for lower moisture, 0.5–1%. The narrowing of the range is especially noticeable at 9% and 11% moisture contents. On the other hand, for a sample moisture of 3%, the intensity variation is the greatest. At the same time, for this moisture, differences in intensity are revealed between samples with larger sizes and those smaller than 0.5 mm (Figure 18).

In the most finely sized material with a moisture content of 3%, particle agglomerates appear (Figure 18a). They are formed when the material is shaken, e.g., when sliding from the pipeline to the working area of the camera. This phenomenon still occurs for 5% moisture, whereafter the water content of the sample is too high for these agglomerates to continue to exist. This phenomenon is characteristic only for the finest fraction; for particles of larger sizes, it does not occur. Of course, it can be reduced by forming the sample in the working area of the camera immediately before taking the image. Namely, this forming may be to flatten the sample, thereby removing any shadows visible in the image.

**Figure 19:**
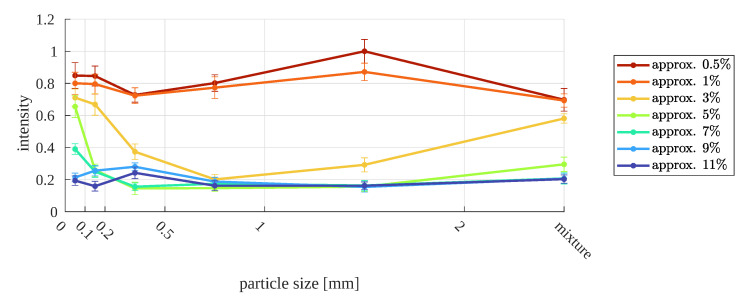
Image median intensity as function of particle size, for different relative moistures.

The influence of 3% moisture is plotted in Figure 19. It is visible especially for particles bigger than 0.2 mm, where intensity values are anomalous compared to intensities acquired for other moisture levels. This is probably due to the admixtures in copper ore (Figure 18), which became particularly apparent when washed with 3% water addition.

**Figure 20:**
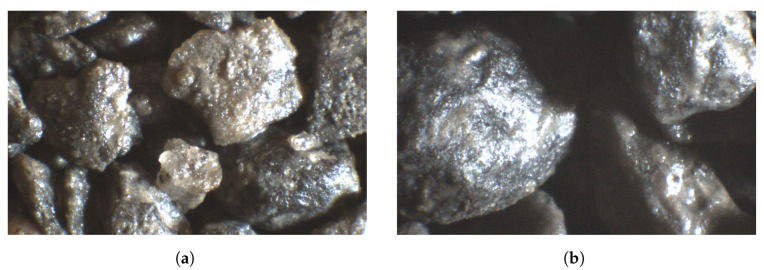
Macro images of samples: (**a**) 0.5–1 mm with 7% moisture; (**b**) 1–2 mm with 5% moisture.

In Figure 18c,d, shiny particles are very visible, which becomes apparent only in higher moisture due to washing of the copper ore particles by water. Undoubtedly, these are admixtures in copper ore—in the form of limestone, for example. This phenomenon can be seen well in macro images (Figure 20). The appearance of water on the surface of particles changes the light reflectance, which, due to the irregular shape of the particles, affects the contrast of the image, and at the same time extracts other impurities from the tested samples. This phenomenon, of course, occurs with practically every particle size and only with larger particle sizes it clearly affects the results.

**Figure 21:**
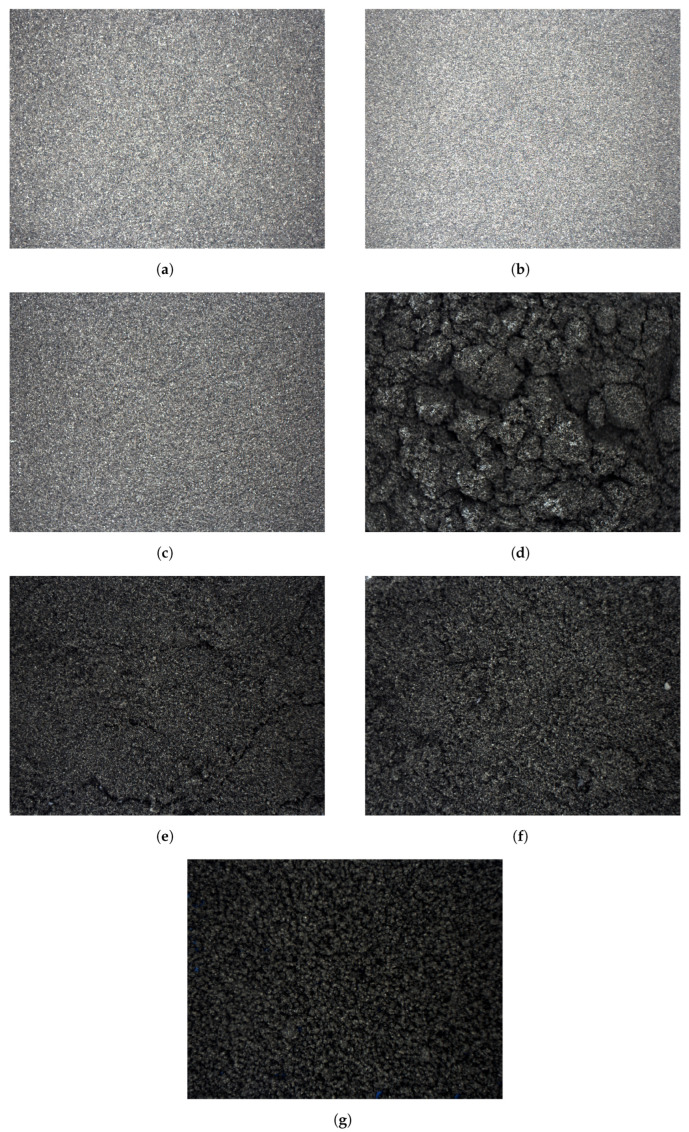
Images of 0.1–0.2 mm material sample with moisture contents of: (**a**) 0.5%, (**b**) 1%, (**c**) 3%, (**d**) 5%, (**e**) 7%, (**f**) 9%, and (**g**) 11%.

At higher moisture values of 7–11%, only a slight change in intensity can be seen, which may also be problematic for moisture modeling at selected particle sizes. The images of the subsequent moisture levels for a 0.1–0.2 mm sample are presented in Figure 21. For the mentioned moisture values, it is practically impossible to find a unique relationship between moisture and image parameters and thus distinguish between moistures in this range. This is contrary to the 0.5–7% moisture range, where there are large differences between intensity values. In this case, linear function modeling can be used only for a small range of moistures, i.e., 0.5–1% (Figure 19); in the wide range 0.5–7%, it would produce large error. Thus, the relation in this range can be fitted, e.g., with spline or polynomial functions, which take into account a dramatic jump in intensity for a moisture content of 3%. This approach requires obtaining information on the particle size distribution of the tested sample, e.g., using algorithms [23,24]. Only after the particle size is known is it possible to automatically select the model for a specific particle size, especially when the moisture must be estimated in online processing directly on site.

**Figure 22:**
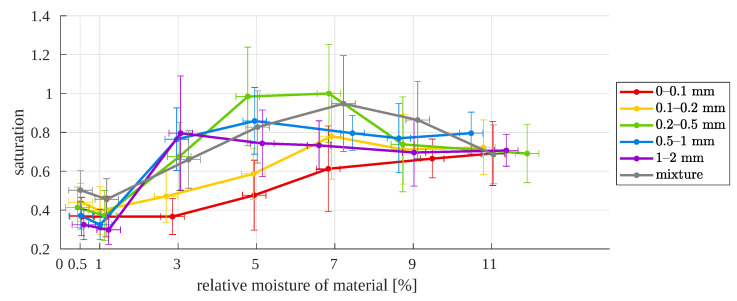
Image median saturation as a function of relative moisture for different particle sizes.

The conducted experiments allowed us to confirm the usefulness of visual analysis for estimating the moisture of copper ore. However, on the one hand, it is necessary to take into account the potential drying of the sample and the formation of the sample. On the other hand, relying only on the characteristics resulting from the color space analysis may not be sufficient for moisture values of 7–11%, where the intensity value is similar for several particle sizes. This problem is not solved even when taking into account the saturation from HSL space (Figure 22). Saturation changes are not always monotonic with an increase in moisture, and the saturation errors are greater than those of intensity. These make saturation a worse indicator of material moisture. Finally, there is the need for particle size estimation first. From this, a typical cascade system will be obtained: particle size detection as distribution in the sample, linear/spline/polynomial model selection, and moisture estimation.

It should be noted that this method is an approximation of the moisture value and numerous phenomena should be taken into account, such as brilliance, shadows, particle agglomerates, and the drying of the sample. However, the advantage of this method is that it allows one to quickly assess the moisture directly in the copper ore grinding process. The only limitation is the need to prepare a model in advance, describing intensity as a function of material moisture. Output of the inverse model can be used as approximate moisture value.

**Figure 23:**
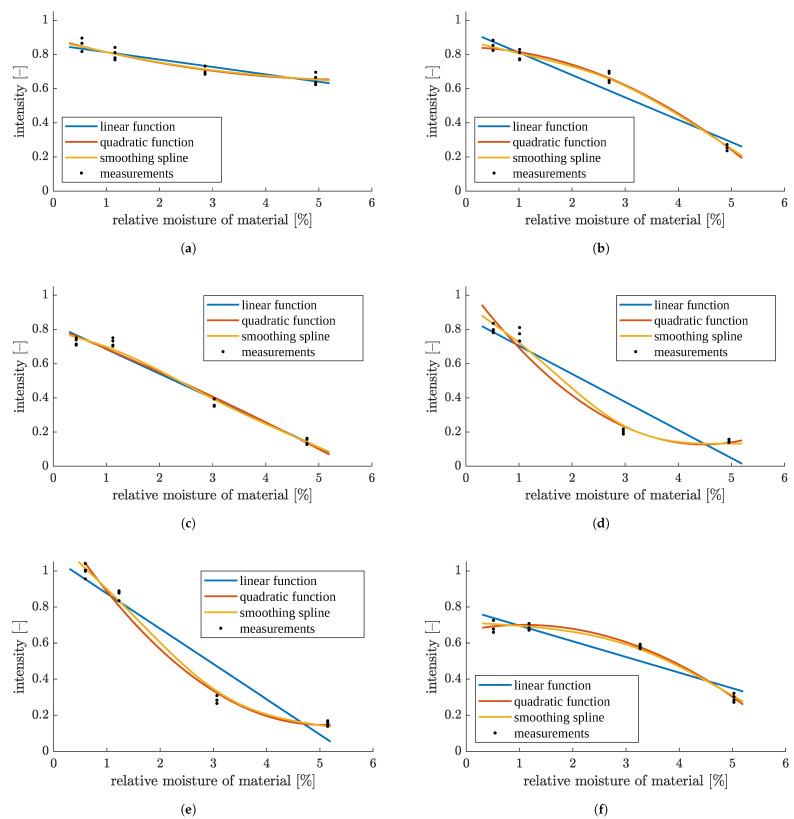
Image median intensity as a function of material moisture, with linear, quadratic and smoothing spline fitting for particles of size: (**a**) <0.1 mm, (**b**) 0.1–0.2 mm, (**c**) 0.2–0.5 mm, (**d**) 0.5–1 mm, (**e**) 1–2 mm and (**f**) mixture.

Some models of intensity vs. moisture relationship are proposed in Figure 23. The corresponding quantitative quality indices are listed in Table 1. More details are given in Appendix B, including procedures used for the estimation of model parameters, and equations for the quality indices.

The simplest case would be a linear function model. However, if moisture is in the range 0.5–5%, then usually quadratic or smoothing spline fitting produces much more accurate results compared to linear fitting (Figure 23). Of course, the models are based on raw data, which contain anomalies for 3% moisture, so these are transferred to the model. It is also important that each of the models has a unique shape, but unfortunately the intensity and moisture values for many of them coincide in several ranges of moisture, which again suggests the need for the earlier detection of the particle size in the sample.

The numeric data in Table 1 confirm the visual observations. Usually, straight-line models are noticeably more poorly fitted than higher-order models. Spline models have rather similar root mean squared errors (RMSE) as quadratic models, and similar or slightly better coefficients of determination R${}^{2}$ and R${}_{\mathrm{adj}}^{2}$. All coefficients of determination are valued above 0.8, and most of them are well above 0.9, which indicates strong statistical correlation between image intensity and material moisture.

Inverting the above models allows estimated values of material moisture content to be produced based on the median intensity of the image. Such inverse models were validated with the leave-one-out technique [46]. This meant that the models were created and verified on two separate sets of images, which resulted in a reliable assessment of model performance for new data (new images). Namely, each image in turn was treated as a single-item test set, and the others were used to estimate the parameters of polynomial and spline models of intensity vs. moisture again. Then, for each model, the moisture corresponding to the normalized median intensity of the test image was returned as the estimated material moisture. Operating range of the method was assumed as 0–5.5% moisture, which means that any possible results outside of this range were discarded. The returned moisture value was compared with the reference measurement from the moisture analyzer, assumed as the true value. The results of this cross-validation are plotted in detail in Appendix C and summarized in Table 2 and Table 3.

Each inverse model may return a single output moisture (desired situation) value, two results (when the model was non-monotonic in the operating range), or no results (either because of the inherent shape of the function or because inverting the polynomial or spline model only yielded results out of the operating range of moisture values). However, under standard operating conditions, moisture measurements were taken continuously on a stream of material whose features do not change rapidly. Thus, in many cases, recent historical data would indicate a correct single value even if two of them were returned by the algorithm. On the other hand, if the output of an inverse model is out of the operating range of moistures, the result may be coerced to the minimum or maximum of this operating range (whichever is closer) and thus, some usable value will be produced from the image. Still, these are less accurate and less reliable data than when a single output value is received from the model. From Table 2 it follows that quadratic function produced unambiguous results the least often, and moreover, it sometimes encountered the least desirable "no results" situation. The other two model types performed better and were similar to each other. Thus, they should be preferred.

The quality of moisture estimation by inverse models was also assessed quantitatively, with the same measures as previously used with the direct-form models. The median image intensity was the predictor, and material moisture was the response variable. (See Appendix B for equations.) During the calculations, missing model outputs (when zero results were returned) were omitted, and the worse of two errors was included when duplicate model outputs were produced. Fit quality indices are presented in Table 3.

RMSE values reached no more than 0.8% moisture, which is less than 15% of the assumed measurement range of up to 5.5% moisture. This indicates that the proposed method gives satisfactory results for approximate moisture measurements in this value range. The worst values of all quality indices occur for the finest fraction, for 0.5–1 mm fraction, and for mixed-size material. For the other three individual fractions, RMSE is even twice lower, and coefficients of determination are also high—mostly around 0.95.

## 5. Conclusions

In this research, we proposed a novel method for the contactless approximate measurement of copper ore moisture, to be used, e.g., in industrial installations. The experiments involved five size fractions of finely ground copper ore, with particles sized 0–2 mm, plus a mixture of these fractions. Relative moisture (mass percentage) of the tested material spanned a wide range, 0.5–11%. Vision and thermovision images of moist copper ore were taken and analyzed.

The presented approach was to analyze the intensity of a digital image in visible light, by means of conventional image processing techniques and modeling. In conclusion, this is a method that allows for the rough estimation of material moisture content for moisture in the range of 0.5–5%. This may be an alternative to classical methods of moisture measurement, especially if image processing is already used for the assessment of other material features (then, moisture could be estimated effortlessly from the same images). For moisture content higher than 5%, there is practically no change in image intensity with the increase in moisture.

There is a certain regularity in data obtained from infrared images, especially when using a macro lens. Unfortunately, no monotonic relationship was observed between the mean temperature determined from thermograms and the material moisture, nor between mean temperature and particle size with moisture being a parameter. It seems that infrared in the long wavelength range cannot be used if the method is based on temperature measurement. However, in further research, the image processing algorithms could be investigated on the thermograms. In the case of the thermography approach or in general, the infrared measurement of moisture, the investigations with NIR (Near InfraRed, 0.78–1.4 µm wavelength) and SWIR/MWIR (Short/Medium Wavelength InfraRed, respectively 1.4–3 or 3–5 µm wavelength) are planned in the future. However, this requires the expansion of the measurement set-up with new cameras operating in these wavelength ranges.

## Figures and Tables

**Table 1:** Goodness of fit of estimated image intensity (output of proposed models) to true intensity (calculated from the images). Quality indices calculated according to Appendix B.

Measure	Model Type	Size Fraction					
		0–0.1 mm	0.1–0.2 mm	0.2–0.5 mm	0.5–1 mm	1–2 mm	Mixture
number of coefficients ^1^	linear	1	1	1	1	1	1
	quadratic	2	2	2	2	2	2
	spline	2.11	2.13	2.09	2.13	2.11	2.09
RMSE	linear	0.032	0.053	0.040	0.11	0.11	0.056
	quadratic	0.028	0.026	0.039	0.057	0.047	0.020
	spline	0.032	0.028	0.035	0.046	0.041	0.025
R${}^{2}$	linear	0.842	0.948	0.974	0.874	0.912	0.883
	quadratic	0.877	0.987	0.975	0.965	0.983	0.985
	spline	0.876	0.988	0.984	0.982	0.990	0.982
R${}_{\mathrm{adj}}^{2}$	linear	0.831	0.944	0.972	0.865	0.906	0.875
	quadratic	0.858	0.985	0.971	0.960	0.980	0.983
	spline	0.856	0.986	0.982	0.979	0.988	0.979

^1^ Excluding free coefficient. This is variable *k* in Equation (A5).

**Table 2:** Number of cases (out of total 16 images per size fraction) when the inverse model tested in leave-one-out manner returned: no outputs at all (*0*); only some out-of-range outputs which were then coerced to a single value (*coer.*); single output (*1*); two outputs (*2*).

Size Fraction	Linear Function				Quadratic Function				Smoothing Spline			
	*0*	*coer.*	*1*	*2*	*0*	*coer.*	*1*	*2*	*0*	*coer.*	*1*	*2*
0–0.1 mm	0	3	13	0	2	1	13	0	0	3	13	0
0.1–0.2 mm	0	1	15	0	2	0	14	0	0	1	15	0
0.2–0.5 mm	0	0	16	0	0	0	16	0	0	0	16	0
0.5–1 mm	0	0	16	0	0	0	12	4	0	0	16	0
1–2 mm	0	0	16	0	1	0	14	1	0	0	16	0
mixture	0	3	13	0	4	0	10	2	0	2	14	0

**Table 3:** Goodness of fit of estimated moisture (output of inverse models) to true moisture (reference measurements). Quality indices calculated according to Appendix B.

Measure	Model Type	Size Fraction					
		0–0.1 mm	0.1–0.2 mm	0.2–0.5 mm	0.5–1 mm	1–2 mm	Mixture
RMSE	linear	0.72	0.45	0.31	0.74	0.61	0.65
	quadratic	0.72	0.35	0.33	0.62	0.38	0.80
	spline	0.70	0.32	0.32	0.60	0.33	0.66
R${}^{2}$	linear	0.823	0.932	0.966	0.820	0.880	0.866
	quadratic	0.773	0.957	0.962	0.875	0.951	0.785
	spline	0.833	0.964	0.964	0.882	0.966	0.864
R${}_{\mathrm{adj}}^{2}$	linear	0.810	0.928	0.964	0.808	0.871	0.856
	quadratic	0.732	0.949	0.956	0.856	0.942	0.738
	spline	0.805	0.959	0.958	0.862	0.961	0.842

**Figure A1:**
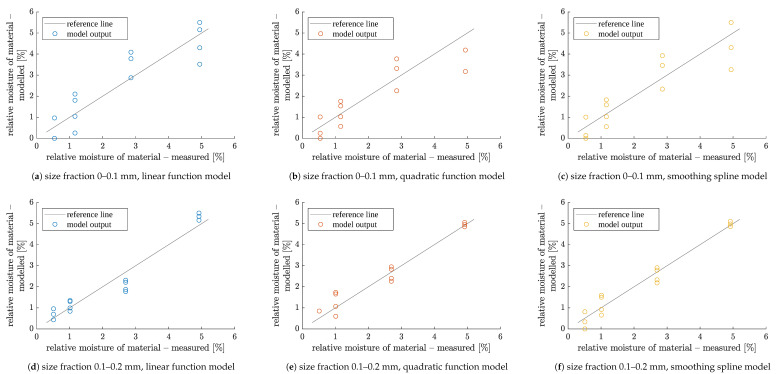

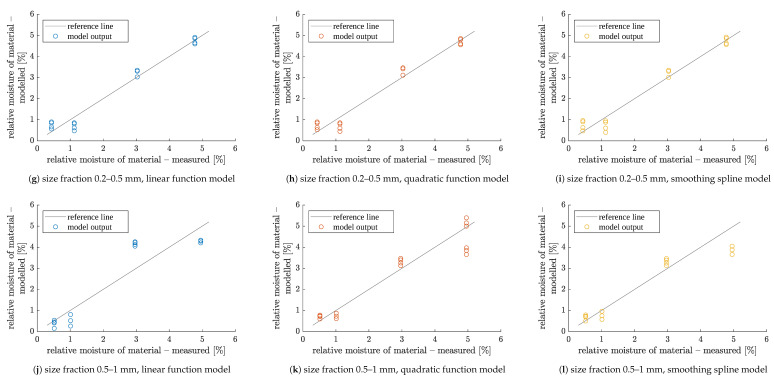

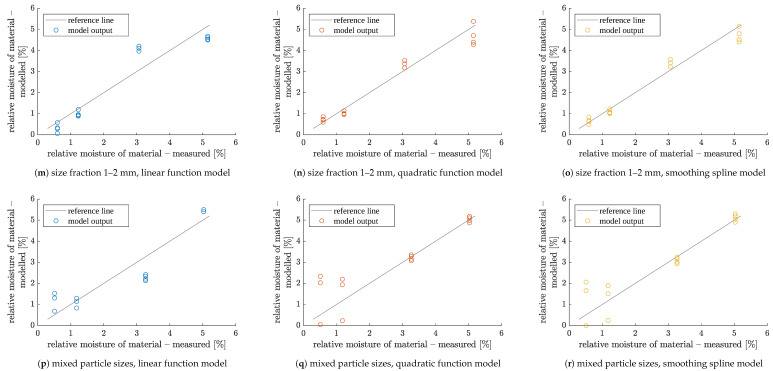
Outputs of inverse models (material moisture vs. image median intensity) as obtained in leave-one-out cross-validation.

**Figure A2:**
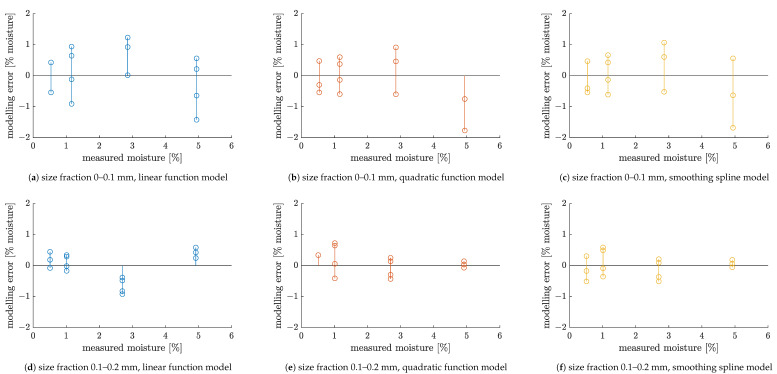

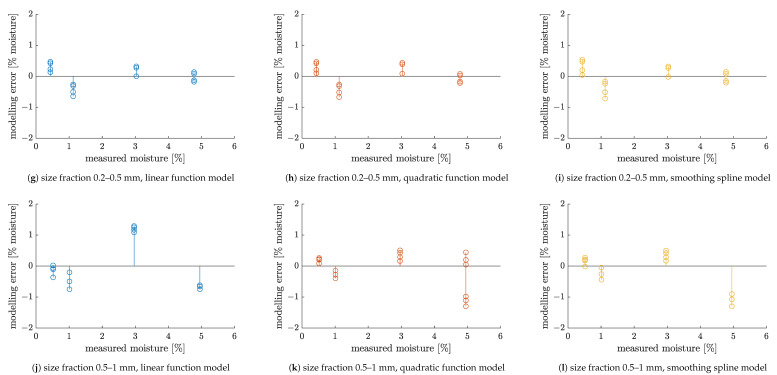

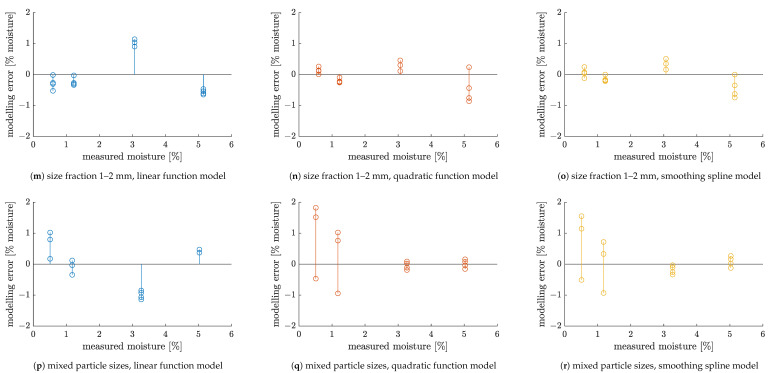
Errors of inverse models (material moisture vs. image median intensity) as obtained in leave-one-out cross-validation.

## Data Availability

The data presented in this study are available in the supplementary materials.

## Supplementary Materials

The following supporting information can be downloaded at https://www.mdpi.com/article/10.3390/s23031220/s1, Table S1: Exact values of relative moisture of the material (mass percentage), measured with moisture analyzer—experiment series 1 (with macro images); Table S2: Exact values of relative moisture of the material (mass percentage), measured with moisture analyzer—experiment series 2 (with normal images); Table S3: Mean surface temperature [deg. C] from thermovision images; average value from all thermograms for given size fraction and moisture level; experiment series 1 (macro images); Table S4: Mean surface temperature [deg. C] from thermovision images; average value from all thermograms for given size fraction and moisture level; experiment series 2 (normal images); Table S5: Mean surface temperature [deg. C] from thermovision images; individual value per thermogram; experiment series 1 (macro images); Table S6: Mean surface temperature [deg. C] from thermovision images; individual value per thermogram; experiment series 2 (normal images); Table S7: Normalized values of median intensity of vision images; average value from all four images for given size fraction and moisture level; normal images (standard lens); Table S8: Non-normalized values of median intensity of vision images; average value from all four images for given size fraction and moisture level; normal images (standard lens); Table S9: Normalized values of median intensity of vision images; individual value per image; normal images (standard lens); Table S10: Non-normalized values of median intensity of vision images; individual value per image; normal images (standard lens); Table S11: Normalized values of median saturation of vision images; average value from all four images for given size fraction and moisture level; normal images (standard lens); Table S12: Non-normalized values of median saturation of vision images; average value from all four images for given size fraction and moisture level; normal images (standard lens); Table S13: Normalized values of median saturation of vision images; individual value per image; normal images (standard lens); Table S14: Non-normalized values of median saturation of vision images; individual value per image; normal images (standard lens).

## Appendix A. Error Bars in the Plots

The main result plots, showing intensity, saturation or temperature values relative to material moisture or particle size, contain error bars. Their lengths were determined as follows:

1. Moisture content: error is ±0.3% moisture.
  This is expanded uncertainty of copper ore moisture measured with the mentioned moisture analyzer [19]. This value accounts for non-homogeneous sample, which might have occurred for very damp material, especially when coarser sized. In the plots, the errors were saturated at 0% moisture, that is, the moisture value including errors was never lower than 0%—because of course, such value would be physically unrealizable.
2. Particle size: errors are not indicated in the graphs.
  The horizontal coordinate of each plotted data point was assumed as the middle of the appropriate particle size range. The choice was somehow arbitrary, but reasonable for illustrative purposes. Such nature of the plotted value made any error bars rather artificial and not much useful, so they were not drawn.
  Perhaps a more detailed illustration would be to use the mean (or median) particle size in the sample. However, this would require a higher-resolution particle size distribution, which was not available in this experiment, and any approximations were difficult to make. Moreover, the sieving process itself is burdened with some inaccuracies—there is always some amount of under- and oversized particles. This would need to be included in the estimated error, making the calculations even more complex. Taking into account the preliminary character of the conducted research, the total effort was assessed as not feasible.
3. Mean surface temperature: errors range from ±0.14 °C to ±2.7 °C, depending on the data point. These are expanded uncertainties combined from type A and B components.
  Type A uncertainty component $u_{A}$ at each data point is the sample standard deviation of values for all thermograms captured for the same material sample (the data point itself shows the mean value of all these thermograms). Type B uncertainty component $u_{B}$ is 50 mK (or 0.050 °C) for the used camera. The expanded uncertainty *U* is then calculated individually for each point in the plot, as:
  (A1) $$U=2\cdot \sqrt{u_{A}^{2}+u_{B}^{2}}\;,$$
  and drawn as error bars along the temperature axis, in both directions.
4. Normalized median intensity of vision images: errors range from 0.018 to 0.081, depending on the data point and direction (below/above the data point). These are again the expanded uncertainties of the measurement (A1).
  Type A component is in this case the sample standard deviation from the four normalized intensity values at each moisture level and particle size. Type B components follow from the assumption that error of raw intensity values in the image was ±1 gray level, with possible intensity values ranging from 0 to 255. Such assumption follows from the format of image files. This ±1 error applies to both the intensities *I* of individual plot points and to their maximum $I_{\max}$, which was used for data normalization (each plotted data point was $I/I_{\max}$). So, the infimum and supremum of each data point were calculated as:
  (A2) $$I_{\inf}=\frac{I-1}{I_{\max}+1}\;;\;I_{\sup}=\frac{I+1}{I_{\max}-1}\;.$$
  Consequently, $u_{B}$ values reach $\left(I-I_{\inf}\right)$ below a data point and $\left(I_{\sup}-I\right)$ above the data point.
5. Normalized median saturation of vision images: errors range from 0.069 to 0.29, depending on the data point and direction (below/above the data point).
  Saturation errors were calculated in the same way as intensity errors. Here, value used for normalization (i.e., maximum of the plotted saturation values) was smaller than in the case of intensities, so the type B components are bigger this time.

It should also be noted that any statistical calculations in this experiment were based on images of the same physical sample. For full assessment of errors, and also for most accurate estimation of model curves, the same experiment should be repeated several times, to produce a few independent material samples for each size fraction and moisture level. However, such experiments would be much time-consuming and they were not necessary for the preliminary research.

## Appendix B. Models of Image Median Intensity vs. Material Moisture

This section details the models presented in Figure 23. They represent the relationship between median image intensity and material moisture, for relative moistures up to 5%. For more damp material, changes in image intensity were not significant enough to be used for moisture estimation (see Figure 17).

The models identified here were linear and quadratic functions, and smoothing splines. Third order polynomials were also tested, but they produced excess curvature of the output for the intermediate values between the tested moistures. The models should be treated as approximate. To derive more accurate estimates, the experiment could be repeated several times for each tested moisture level and size fraction. Also, some additional moisture values could be tested in the 1–5% range.

First and second order polynomial models were identified with ordinary least squares method. Errors in predictor variable (moisture content) were identical for all data points; errors in response variable (intensity) were similar between data points and very small in relation to the whole range of intensities for each data series. Thus, it was not necessary to use weighted least squares method that take into account the errors in both variables—the difference in the resultant model coefficients would be negligible. Additionally, cubic smoothing spline model was identified, using built-in MATLAB function fit [47]. The parameter p∈[0,1] that defines the trade-off between total smoothening (p=0) and exact following of the data points (p=1) was selected automatically. For the given datasets, its value was around 0.8–0.85.

As seen from the graphs in Figure 23, accuracy of straight line model is reasonable only for the second fine fraction (Figure 23b), somehow debatable for the middle-sized fraction and mixture (Figure 23c,f), and not sufficient for coarser particles (Figure 23d,e) or for the finest ones (Figure 23a), where the error in intensity estimation is relatively big compared to the whole range of intensities for this dataset. Quadratic polynomial or spline models perform much better and seem suitable for all tested cases. Fit quality is similar for both these model types, with spline model being slightly better for lower moistures (0.5–3%) in coarse material (Figure 23d,e). Also, in some cases splines produce more reasonable output (less diverging from the neighbouring data points) near to the extreme predictor values, that is, close to 0.5% and 5% moistures. This is observed in Figure 23c–e.

Numerical indices describing the models and their goodness of fit to the data are collected in Table 1. The following measures are presented, assuming the notation: *N*—number of data points in the dataset, $y_{i}$—measurements of response variable (here: intensity), ${\hat{y}}_{i}$—samples of model output, $\bar{y}$—mean value of response variable, *k*—number of model parameters (excluding the free coefficient, if present):

- root mean squared error:
  (A3) $$\mathrm{RMSE}=\sqrt{\frac{1}{N}\sum_{i=1}^{N}{\left(y_{i}-{\hat{y}}_{i}\right)}^{2}}\;;$$
- coefficient of determination:
  (A4) $$R^{2}=1-\frac{\sum_{i=1}^{N}{\left(y_{i}-{\hat{y}}_{i}\right)}^{2}}{\sum_{i=1}^{N}{\left(y_{i}-\bar{y}\right)}^{2}}\;;$$
- adjusted coefficient of determination:
  (A5) $$R_{\mathrm{adj}}^{2}=1-\left(1-R^{2}\right)\cdot \frac{N-1}{N-1-k}\;.$$

## Appendix C. Results of Model Cross-Validation

Models derived in Appendix B were inverted to produce material moisture estimates for a given input intensity of an image. Leave-one-out cross-validation was used to assess the performance of all models, as explained in Section 4.2. Acquired outputs of inverse models are plotted in Figure A1 and their errors in relation to reference values are shown in Figure A2.
